# Assessment of DNA methylation in porcine immune cells reveals novel regulatory elements associated with cell-specific gene expression and immune capacity traits

**DOI:** 10.1186/s12864-022-08773-5

**Published:** 2022-08-11

**Authors:** Ryan J. Corbett, Andrea M. Luttman, Juber Herrera-Uribe, Haibo Liu, Nancy E. Raney, Jenna M. Grabowski, Crystal L. Loving, Christopher K. Tuggle, Catherine W. Ernst

**Affiliations:** 1 Genetics & Genome Sciences Graduate Program, Michigan State University, East Lansing, MI USA; 2 Department of Animal Science, Iowa State University, Ames, IA USA; 3 Department of Animal Science, Michigan State University, East Lansing, MI USA; 4 USDA ARS National Animal Disease Center, Ames, IA USA

**Keywords:** DNA Methylation, WGBS, Immune Cells, Pig

## Abstract

**Background:**

Genetics studies in the porcine immune system have enhanced selection practices for disease resistance phenotypes and increased the efficacy of porcine models in biomedical research; however limited functional annotation of the porcine immunome has hindered progress on both fronts. Among epigenetic mechanisms that regulate gene expression, DNA methylation is the most ubiquitous modification made to the DNA molecule and influences transcription factor binding as well as gene and phenotype expression. Human and mouse DNA methylation studies have improved mapping of regulatory elements in these species, but comparable studies in the pig have been limited in scope.

**Results:**

We performed whole-genome bisulfite sequencing to assess DNA methylation patterns in nine pig immune cell populations: CD21^+^ and CD21^−^ B cells, four T cell fractions (CD4^+^, CD8^+^, CD8^+^CD4^+^, and SWC6γδ^+^), natural killer and myeloid cells, and neutrophils. We identified 54,391 cell differentially methylated regions (cDMRs), and clustering by cDMR methylation rate grouped samples by cell lineage. 32,737 cDMRs were classified as cell lowly methylated regions (cLMRs) in at least one cell type, and cLMRs were broadly enriched in genes and regions of intermediate CpG density. We observed strong correlations between differential methylation and expression across immune cell populations, with cell-specific low methylation disproportionately impacting genes exhibiting enriched gene expression in the same cell type. Motif analysis of cLMRs revealed cell type-specific enrichment of transcription factor binding motifs, indicating that cell-specific methylation patterns may influence accessibility by trans-acting factors. Lastly, cDMRs were enriched for immune capacity GWAS SNPs, and many such overlaps occurred within genes known to influence immune cell development and function (*CD8B, NDRG1*).

**Conclusion:**

Our DNA methylation data improve functional annotation of the porcine genome through characterization of epigenomic regulatory patterns that contribute to immune cell identity and function, and increase the potential for identifying mechanistic links between genotype and phenotype.

**Supplementary Information:**

The online version contains supplementary material available at 10.1186/s12864-022-08773-5.

## Introduction

The porcine immune system plays critical roles in combatting infectious diseases, including those prevalent in production systems [1]. As in other mammals, pig immunity is conferred by two defense systems – innate and adaptive immunity. Innate immunity involves many barrier systems and immune cells including myeloid-derived macrophages, dendritic cells, and granulocytes as well as lymphoid-derived natural killer (NK) cells. Adaptive immunity refers to acquired immunity and is governed by a diverse catalogue of lymphoid-derived B and T cells. In addition to adaptive immune cells phenotyped in humans and mice, pigs exhibit an overrepresentation of specific T cell subsets in peripheral blood. These include CD8+CD4+double-positive (DP) T cells, which express CD8αα as opposed to CD8 αβ on conventional cytotoxic T cells and are functionally characterized as a memory T cell population with MHC-II restriction [2–4]. In addition, pigs are considered a gamma-delta (γδ) high species with frequencies of circulating γδ T cells of up to 30% [5]. γδ T cells are defined by the expression of T cell receptors (TCR) composed of gamma and delta subunits, as opposed to the alpha and beta chain TCR of more conventional αβ T cells. γδ T cells play roles in both the innate and adaptive immune response [6] and differential expression of CD2 and CD8α on γδ T cells is indicative of function [7]. One reagent developed to label porcine γδ T cells, SWC6, labels most, but not all circulating γδ T cells in pigs [8]. Increased functional characterization of pig leukocytes—and in particular pig-enriched T cell populations—will inform understanding of their unique roles in immune response pathways.

Genetics studies in the pig have enhanced the discovery of DNA variants associated with immune-related traits: to date 3,236 and 619 QTL for immune capacity and disease susceptibility traits, respectively, have been submitted to the Pig QTL Database (https://www.animalgenome.org/cgi-bin/QTLdb/SS/index). Furthermore, comparative genomics studies revealed greater similarity between human and pig immune genes relative to human and mouse genes [9], making the pig a promising biomedical model to study human diseases [10, 11]. However, limited functional annotation of the porcine genome—particularly within regulatory regions—has limited both the search for causative variants influencing disease and production traits in pigs as well as the translational capabilities of the pig as a model organism [12]. To this end, the Functional Annotation of Animal Genomes (FAANG) consortium was initiated to map functional elements in domesticated food animal species through the use of high-throughput sequencing data generated from tissues and cell types of relevance, including those of the immune system [13, 14]. Among the core FAANG assays are many that assess epigenetic modifications that regulate chromatin accessibility and gene expression, including DNA methylation, histone modifications, and long non-coding RNAs [15]. FAANG projects in the pig have begun characterizing epigenetic patterns in immune cell populations, including in LPS- and Poly-I:C-stimulated cells [16–18].

DNA methylation is the most ubiquitous epigenetic modification made to the DNA molecule, and involves the enzymatic addition of a methyl group to the 5-position of cytosine rings to produce 5-methylcytosine. Methylation occurs almost exclusively at CpG dinucleotides in mammals, although CpG-dense regions known as CpG Islands—genomic regions with an observed-to-expected CpG dinucleotide ratio greater than 0.6—are generally unmethylated and are highly prevalent in the promoters of ubiquitously expressed genes. DNA methylation exhibits context-specific associations with gene expression; at gene promoters and enhancers, methylation generally functions to decrease levels of transcription through the alteration of transcription factor (TF) binding sites or the recruitment of transcriptional repressors and chromatin-modifying enzymes [19–21]. Generation of DNA methylomes from diverse cell types across animal species has led to the identification of cell-differentially methylated regions (cDMRs) that have vastly improved understanding of state-specific epigenetic gene regulation. Many such studies have identified strong associations between cell-specific lowly methylated regions (cLMRs) and TF binding motifs as well as GWAS SNPs for relevant traits, highlighting the potential significance of these regions and of DNA methylation in regulating gene and phenotype expression [22–26]. DNA methylation patterns have previously been characterized in mixed porcine immune cell preparations [17, 27]. However, assessment of methylation patterns in specific porcine immune cell types has not been performed, but could provide insight into transcriptional regulation and hence function relevant to pig health.

Here we report the first genome-wide DNA methylation study in porcine granulocytes (primarily neutrophils) and eight sorted immune cell populations from peripheral blood: myeloid cells (monocytes/DCs), NK cells, two B cell fractions (CD21^+^ and CD21^−^) and four T cell fractions (CD8^+^, CD4^+^, CD8^+^CD4^+^, and SWC6γδ^+^). Using whole-genome bisulfite sequencing (WGBS), we identified cell-differential DNA methylation patterns strongly associated with enriched gene expression and TF binding sites governing cell specificity. Furthermore, we report cDMRs overlapping previously identified GWAS SNPs for immune-related traits, suggesting they may play important roles in regulating gene expression that impacts phenotypic variation. These data massively improve functional annotation of the porcine immunome and provide unique insight into epigenetic gene regulation in understudied immune cell populations.

## Materials & methods

### Blood collection and separation

Blood was collected from two ~ 6-month-old purebred Yorkshire male pigs. Pigs were housed in BSL2 rooms at the National Animal Disease Center (Ames, IA) and all animal procedures were performed in compliance with and with approval by the Institutional Animal Care and Use Committee. Blood was drawn and peripheral blood mononuclear cells (PBMCs) were isolated as described in Herrera-Uribe et al. 2021 [18]. For granulocyte isolation, blood was collected in BD Vacutainer ACD solution A tubes and subjected to dextran sedimentation using 6% Dextran/0.9% NaCl solution at room temperature for 45–60 min. The supernatant was transferred to a conical tube and centrifuged for 12 min at 300 RCF and 4° C with low brake. The supernatant was discarded and the pellet was resuspended in ACK Lysing buffer per manufacturer’s instructions (Thermo Fisher) to lyse contaminating red blood cells. The cell suspension was centrifuged at 300 RCF for 5 min to pellet intact granulocytes. After removal of lysed RBCs in the supernatant, the pellet was resuspended in phosphate buffered saline (PBS) and overlayed onto Ficoll-Histopaque-1077 (Catalog No.1077, Sigma) and centrifuged for 30 min at 450 RCF at room temperature and low brake. The PBMC cell layer was discarded, and the pellet (containing granulocytes) was further processed. Granulocytes (primarily neutrophils) were rinsed with 4 ml PBS and centrifuged at 450 RCF for 5 min. The resulting pellet was resuspended in 2 mL HBSS and viable cells were enumerated using the Count and Viability Assay Kit on the MUSE® cell analyzer system (Millipore). Cells were lysed using RLT buffer (Qiagen) and lysed material was stored at -80° C until further use.

### Sorting PBMCs into specific immune populations

PBMCs underwent Magnetic- and Fluorescence-Activated Cell Sorting (MACS/FACS) as previously described [18]. Briefly, PBMCs were incubated with biotin labeled anti-porcine CD3ε (PPT3, Washington State University Monoclonal Antibody Center) and separated into CD3ε -positive and CD3ε -negative fractions on LS columns. Each fraction was further separated by FACS into four subpopulations (total of 8 cell populations) based on extracellular markers. Isolated subpopulations in the CD3ε-positive fraction included SWC6γδ + T cells (**SWC6gdT**; CD3ε^+^SWC6^+^), CD4^+^ T cells (**CD4T**; CD3ε^+^SWC6^−^CD8α^−^CD4^+^), CD8^+^ T cells (**CD8T**; CD3ε^+^SWC6^−^CD8α^+^CD4^−^), and CD8^+^CD4^+^ T cells (**CD4CD8T**; CD3ε^+^SWC6^−^CD8α^+^CD4^+^). Isolated subpopulations from the CD3ε-negative fraction included myeloid leukocytes (**myeloid**; CD3ε^−^CD172α^+^CD8α^−^), NK cells (**NK**; CD3ε^−^CD172α^−^CD8α^+^), CD21^+^ B cells (**CD21pB**; CD3ε^−^CD172α^−^CD8α^−^CD21+), and CD21^−^ B cells (**CD21nB**; CD3ε^−^CD172α^−^CD8α^−^CD21^−^). A fraction of each sorted population was lysed with RLT buffer (Qiagen) and preserved material was stored at -80° C until further use.

### DNA isolation and bisulfite sequencing

DNA was isolated from cells using the Qiagen AllPrep DNA/RNA Minikit and quantified using a Qubit fluorometer. Prior to library preparation, sample DNA was spiked with unmethylated lambda phage DNA (Promega) at a concentration of 5 ng lambda DNA/1 μg sample DNA. DNA was fragmented to approximately 350 bp using a Covaris M220 Sonicator, and bisulfite-converted using the Zymo EZ DNA Methylation-Gold Kit according to manufacturer’s instructions (Zymo Research). Bisulfite sequencing libraries were prepared using the Accel-NGS Methyl-Seq DNA Library Kit and Methyl-Seq Combinatorial Dual Indexing Kit (Swift Biosciences). Completed libraries were quantified and QC’ed using Qubit dsDNA HS and Agilent 4200 TapeStation HS DNA1000 assays, respectively.

Sequencing libraries were divided into three pools of six libraries, and WGBS was performed on each pool across three flow cell lanes on an Illumina HiSeq 4000 instrument in 2 × 150PE format using HiSeq 4000 reagents. A PhiX control DNA library was spiked into each lane at 10% of the total to account for the unbalanced base composition inherent in Methyl-Seq libraries. Base calling was done by Illumina Real Time Analysis (RTA) v2.7.7 and output of RTA was demultiplexed and converted to FastQ format with Illumina Bcl2fastq v2.19.1.

### WGBS bioinformatics analyses

WGBS libraries were trimmed of technical sequences and low-quality bases using Trimmomatic v.0.39 [28]. Forward and reverse reads were subjected to removal of the first 10 and 15 bases, respectively, according to the Swift Biosciences Library Kit instructions. Reads were further trimmed using the following parameters: ILLUMINACLIP: < adapter sequence > :2:30:10 LEADING:25 TRAILING:25 AVGQUAL:20 MINLEN:30. Trimmed reads were aligned to the *Sus scrofa* reference genome (v11.1) using Bismark v.0.18.1 [29]. The bismark_genome_preparation command was used to prepare an *in-silico* bisulfite-converted bowtie2 index using default parameters. WGBS paired-end alignment was performed using the parameters: -X 1000 –score_min L,0,-0.6. Unmapped forwards reads were merged with forwards reads left unpaired following trimming and aligned using the same parameters. All libraries were also aligned to the lambda genome using default parameters, and the percentage of methylated cytosines among lambda-derived reads was subtracted from 100 to calculate bisulfite conversion efficiency. Aligned WGBS reads were deduplicated using the bismark_deduplicate command, and CpG methylation reports were generated using the bismark_methylation_extractor command with default parameters.

### cDMR and cLMR identification

Genome regional methylation rates were calculated using the *methylKit* R package v.1.8.1 [30]. Briefly, CpG reports from all samples were merged, and genome tiling was performed to calculate average methylation rates for all 1 kb non-overlapping regions in the pig genome. Regions with coverage > 25 in at least 7 cell types per animal were retained for further analysis. For each region *i*, a linear mixed effects model was fitted with methylation rate as a response, and including the fixed effect of cell type and random effect of animal of sample *j*:

$$meth\_rate_{ij}={\;\mu}_i+cell\_type_j+animal_j+\varepsilon_{ij}$$

We assessed the significance of the cell effect using two-way analysis of variance (ANOVA), and regions with a multiple test-corrected cell effect false discovery rate (FDR) < 0.01 were defined as cDMRs. Standardized scores (z-scores) were calculated from methylation rates at each cDMR, and those regions with average cell-type z-score < -1 and mean methylation rate < 75% in a given cell type were further classified as cell lowly methylated regions (cLMRs) for that cell type. cLMRs were annotated using the *genomation* R package v.1.14.0 [31], and cLMR genes were submitted to the Panther database for Gene Set Enrichment Analysis (GSEA) [32, 33].

### Integration of gene methylation and expression data

RNA-sequencing data for the same sorted immune cell populations (*N* = 1 NK cell sample, *N* = 2 all other cell types) from the same animals were previously reported and used in the current analysis [18]. The neutrophil RNA-seq data will be reported in detail elsewhere (Herrera-Uribe, Lim et al., manuscript in preparation). Briefly, transcript abundance was quantified as transcripts per kilobase million (TPM) for all samples, and genes exhibiting cell-enriched expression were identified using the *DESeq2* R package [34]. Briefly, a negative binomial model was fitted for each gene that included the fixed effects of cell type and animal, and expression-enriched genes were defined as those with log2-Fold Change > 1 and FDR < 0.05 in a cell type relative to all other cell types [18]. For each cDMR, we calculated Pearson correlation coefficients between cDMR methylation rates and transcript abundance of overlapping Ensembl-annotated genes across cell populations, further separating cDMRs into those overlapping promoters (< 2 kb from transcription start site), gene bodies, and transcription termination sites (TTSs). For intergenic cDMRs, we calculated correlation coefficients between methylation rates and transcript abundances of the gene with the most proximal transcription start site (TSS) to the cDMR. We compared correlation distributions to those derived from a random sampling of 1 kb regions for each feature and corresponding transcript abundances. To determine the degree of association between low gene methylation and enriched expression, we calculated enrichment *p*-values between lowly methylated genes and expression-enriched genes for each cell type using hypergeometric tests performed by R software.

### Transcription factor binding motif enrichment analyses

We extracted cLMR sequences and sequences from an equal number of random regions for each cell type. cLMR sequences were submitted for Analysis of Motif Enrichment by the MEME Suite [35, 36], using random sequences as controls. Motifs from the JASPAR CORE vertebrates NON-REDUNDANT (*in-vivo* and *in-silico*), UniProbe Mouse, and Jolma2013 Human and Mouse databases [37–39] were scored using the average odds score method, and motif enrichment was calculated using Fisher’s exact test. Motif enrichment clustering was performed using the *pheatmap* R package v.1.0.12 [40].

### GWAS SNP enrichment analysis

GWAS SNPs associated with immune-related and nonrelated traits were downloaded from the Pig QTL Database [41]. We performed hypergeometric tests (base R software) to calculate the enrichment of peak SNPs for select trait classes within cDMRs.

### RT-qPCR analyses

We quantified transcript abundances of genes overlapping cLMRs and previously-reported GWAS SNPs using quantitative reverse-transcriptase polymerase chain reaction (RT-qPCR). Whole blood buffy coats were collected from 7-week-old Yorkshire pigs as part of a previous study and genotyped on the the GeneSeek Genomic Profiler for Porcine HD version 1 commercial BeadChip (Neogen Corporation) [42]. RNA was extracted using TRIzol from buffy coats of an equal number of males and females representing genotypes of SNPs of interest (*CD8B*: rs81371115, 4:g.57971247C > T; *NDRG1*: rs327164077, 4:g.8034723 T > C; *N* = 10–14 animals per genotype). Two μg of RNA was reverse-transcribed using the High Capacity cDNA Reverse Transcription Kit (Applied Biosystems) and cDNA was quantified on a Nanodrop spectrophotometer. *B2M* and *GAPDH* were selected as reference genes due to their observed stable expression in PBMCs [43, 44]. qPCR assays were performed in duplicate on a StepOnePlus Real-time PCR Instrument (Applied Biosystems) using 5 μl cDNA (500 ng total), 1 μl TaqMan Gene Expression Assay (Applied Biosystems Assay Nos. Ss03391669 (*CD8A*) and Ss06890240 (*NDRG1*), Ss03391154_m1 (*B2M*), Ss03374854_g1 (*GAPDH*)), 10 μl, TaqMan Fast Advanced Mastermix (Applied Biosystems), and 4 μl water. Reaction conditions were ﻿50 °C for 2 min and 95 °C for 2 min, followed by 40 cycles of 95 °C for 1 s and 60 °C for 20 s. Delta Cts (dCts) were obtained for each sample by subtracting the geometric mean of the reference gene Cts from the test assay Ct, and analysis of variance and post-hoc pairwise comparisons were performed on average dCts to identify significant differences between genotypes.

## Results

### Porcine immune cells exhibit unique DNA methylation patterns associated with cell-specific co-receptor activation and biological processes

We generated 146-184 M WGBS reads for each immune cell subpopulation, of which 88.3–91.0% aligned to the *S. scrofa* reference genome (Table 1). We observed bisulfite conversion rates > 99% and Pearson correlation coefficients between replicates > 0.8, meeting the standards for WGBS libraries set by ENCODE (https://www.encodeproject.org/data-standards/wgbs/). Average global CpG methylation rates for cell types ranged from 80.1% to 84.3%, with significant differences observed between cell types. Methylation rates of myeloid, CD4T, CD8T, and SWC6gdT cells were significantly higher than those of CD21pB, NK, and CD4CD8T cells. We assessed whether global methylation rates were associated with corresponding expression of DNA methyltransferases (*DNMT*s), and observed a non-significant positive correlation with *DNMT3A* (*r* = 0.469, *p* = 0.057), a slight but not significant negative correlation with *DNMT1* (*r* = -0.249, *p* = 0.336) and a significant negative correlation with *DNMT3B* (*r* = -0.517, *p* = 0.034; Figure S1). When considering the positive association with *DNMT3A* and the negative associations with *DNMT1* and *DNMT3B*, overall *DNMT* abundance was most significantly correlated with CpG methylation (*r* = 0.649, *p* = 5.00E-3), demonstrating that combined *DNMT* expression explained a significant proportion of global methylation variation. Furthermore, we observed that the genes most significantly correlated with global CpG methylation were enriched for DNA replication GO terms (data not shown). We have thus identified methyltransferase and DNA replication genes as significant correlates to DNA methylation levels, suggesting that these processes have a significant impact on methylation signatures in porcine immune cells.

**Table 1:** Summary of Immune Cell WGBS Libraries and Global DNA Methylation

Cell Type	Symbol	Avg. Raw Reads (M)	Mapping Rate (%)	Avg. Clean Reads (M)	Conversion Efficiency (%)	Avg. Global Methylation Rate (%)*	Replicate Correlation
CD4^+^ T	CD4T	174.1	88.3	131.6	99.4	84.3^a^	0.84
SWC6γδ^+^ T	SWC6gdT	168.1	88.6	128.4	99.4	84.1^a^	0.81
Myeloid	Myeloid	146.0	88.8	111.6	99.4	83.5^a^	0.81
CD8^+^ T	CD8T	183.8	88.6	140.0	99.4	83.1^a^	0.81
Neutrophil	Neut	162.6	90.5	122.9	99.4	82.3^ab^	0.80
CD21^−^ B	CD21nB	152.4	88.5	115.6	99.4	82.2^abc^	0.80
CD21^+^ B	CD21pB	150.6	90.3	113.3	99.4	80.3^bc^	0.83
NK	NK	172.8	88.9	133.0	99.4	80.3^bc^	0.80
CD4^+^CD8^+^T	CD4CD8T	170.1	91.0	127.9	99.4	80.1^c^	0.83

^*^Letters indicate statistically significant differences between cell types

**Fig. 1:**
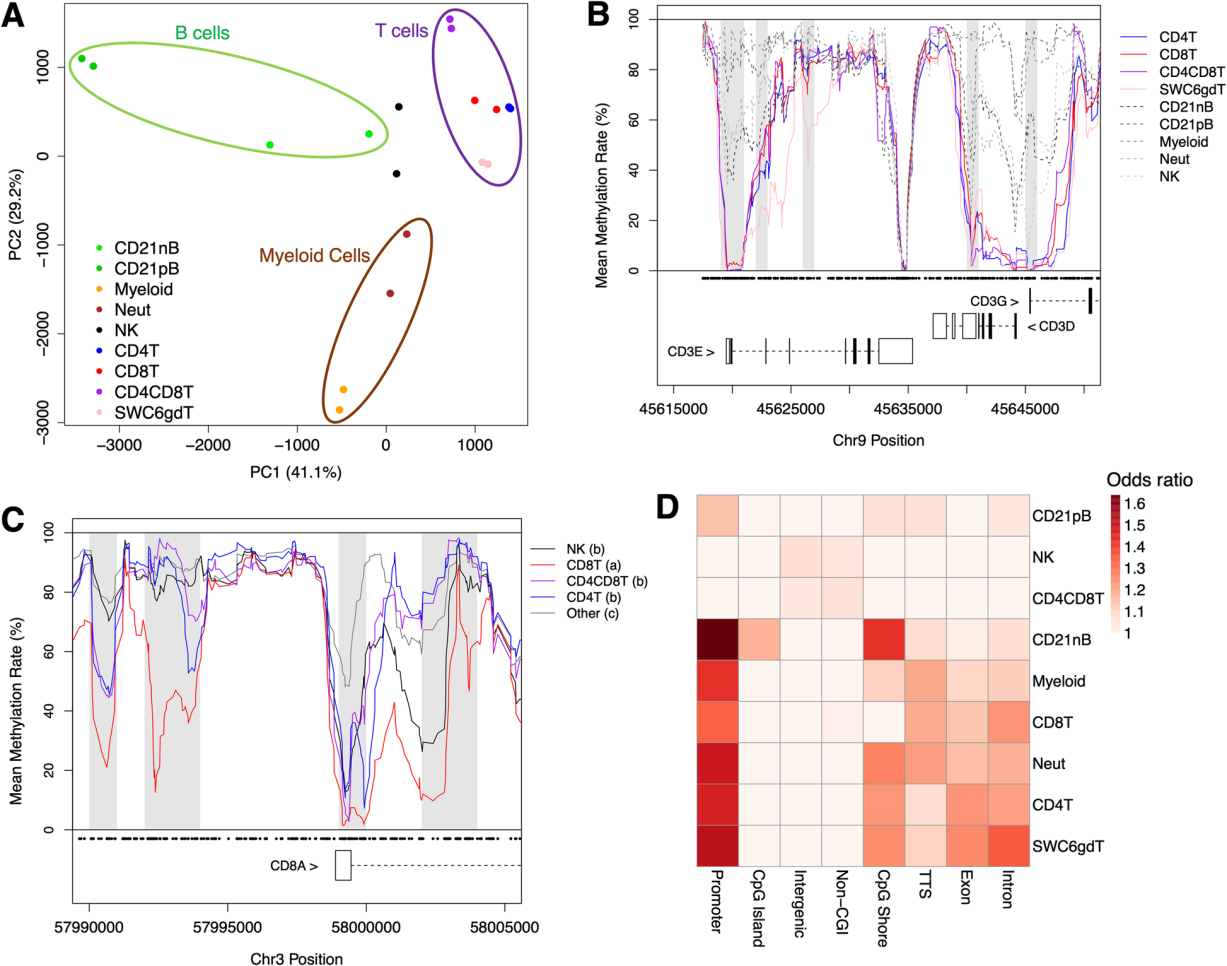
Evidence for lineage-specific immune cell differential methylation. **A** Principal component analysis plot of porcine immune cells based on cDMR methylation rate. **B** Methylation rates across the *CD3* gene locus for T cell populations (solid lines) and other cell types (dashed lines). Gray boxes indicate cDMRs, and black dots indicate CpG coordinates. **C** Methylation rates surrounding the *CD8A* transcription start site for expressing and non-expressing cell types. Letters in legend indicate significant differences in cDMR methylation (gray boxes) between cell types. **D** Heatmap of immune cell lowly methylated region (cLMR) enrichment scores in genomic features. Numbers indicate enrichment *p*-values. cDMR = cell differentially methylated region

We identified 54,391 regions at which methylation rate was significantly associated with cell type, hereby classified as cDMRs (Table S1). A principal component analysis (PCA) revealed that PC1 and PC2 explained 41.1 and 29.2% of cDMR methylation variance, respectively, and clearly separated cell types into those of B, T, and myeloid cell lineages (Fig. 1A). To determine if differential immune cell methylation was occurring within expected genes, we scanned for cDMRs within genes encoding proteins used for cell sorting. We identified multiple cDMRs within the *CD3* multi-gene locus at which methylation in T cells was significantly lower than other cell types (Fig. 1B). We observed additional cDMRs exhibiting hypomethylation within immune cell marker genes *CD19* (in B cells), *SIRPA* (in myeloid cells and neutrophils), *TBX21* (in NK cells), and *CD4* (in CD4T cells and CD4CD8T cells; Figure S2A-D), revealing putative novel sites of gene regulation associated with marker expression. Intriguingly, *CD8A* was unique among marker genes in that methylation patterns were inconsistent with observed *CD8A* mRNA and CD8α protein expression across cell types. Despite not expressing *CD8A* transcript or protein, CD4T cells possessed methylation levels similar to that of CD4CD8T cells throughout the *CD8A* gene locus, most notably in TSS-proximal cDMRs where methylation levels of CD4T were significantly lower than those of other *CD8A* non-expressing cell types (Fig. 1C, S3). These findings suggest that CD4T cells possess a methylation landscape distinct from other CD8α^*low*^ immune cells that may be more permissible to *CD8A* transcription, consistent with the hypothesis that mature porcine CD4^+^ T cells can acquire *CD8A* expression to become CD8CD4 double-positive T cells [45].

To further classify cDMRs based on cell types in which low methylation was observed, we designated all cDMRs with methylation rate < 75% and z-score < -1 in a given cell type as cell lowly methylated regions (cLMRs). A total of 32,737 cLMRs were identified, ranging from 1,196 in CD21nB cells to 13,701 in CD21pB cells, (Table 2, Table S2). We mapped cLMRs to gene features as well as CpG islands (CGIs) and identified feature- and cell-specific enrichment (Fig. 1D). cLMRs in six of the nine immune cell types were most significantly enriched in gene promoters, and to a lesser extent in other gene features (exons, introns, and TTSs) as well as CpG shores. Conversely little to no enrichment was observed in intergenic regions, CGIs, and non-CGIs across cLMRs, suggesting that cell-specific low methylation indicative of gene regulation is more prominent in genomic regions proximal to genes as well as loci of intermediate CpG density. cLMRs in three cell types (CD21pB, NK and CD4CD8T cells) were not enriched for any genomic features, which is in agreement with the observed global hypomethylation of these three immune cell types relative to other cell types. We submitted cLMR-overlapping genes of each cell type for GSEA, and identified uniquely enriched GO terms associated with respective cell functions (Table 3, Table S3). We identified significant enrichment of CD4CD8T cLMR genes for processes related to interferon-gamma production and interleukin-15 signaling, in agreement with known function of CD4CD8T cells in the porcine periphery [2]. These results indicate a strong association between immune cell differential methylation and marker gene expression as well as groups of genes involved in cell-specific biological processes.

**Table 2:** Summary of identified cell lowly methylated regions and overlapping genes

Cell Type	No. cLMRs	No. Unique cLMRs*	No. Genes	No. Unique Genes**
CD21nB	1196	174 (14.5%)	860	28 (3.3%)
CD21pB	13701	9398 (68.6%)	5327	1615 (30.3%)
Myeloid	7959	4640 (58.3%)	3975	916 (23.0%)
Neut	2837	666 (23.5%)	1880	147 (7.8%)
NK	4894	2410 (49.2%)	2268	362 (16.0%)
CD4T	1785	450 (25.2%)	1057	64 (6.1%)
CD8T	1493	375 (25.1%)	901	36 (4.0%)
CD4CD8T	7873	3598 (45.7%)	3412	597 (17.5%)
SWC6gdT	1655	907 (54.8%)	1061	141 (13.3%)

^*^cLMR identified in only one cell type

^**^Gene contains a cLMR in only one cell type

**Table 3:** Cell LMR Gene GO Enrichment

GO Term	No. Genes	Enrichment	FDR
**CD21nB cell LMR Genes**			
Regulation of B cell receptor signaling pathway	9	5.54	1.06E-02
B cell activation	32	3.14	2.17E-05
B cell differentiation	21	2.95	3.62E-03
**Neut LMR Genes**			
Positive regulation of cell motility	107	1.81	1.25E-05
Neutrophil activation involved in immune response	94	1.80	7.59E-05
Neutrophil degranulation	93	1.80	9.29E-05
**CD4T cell LMR Genes**			
Alpha–beta T cell differentiation	16	6.9	1.85E-06
CD4-positive, alpha–beta T cell differentiation	11	6.81	2.24E-04
T-helper cell differentiation	8	6.67	3.95E-03
**CD4CD8T cell LMR Genes**			
Interleukin-15 mediated signaling pathway	8	5.32	1.65E-02
Positive regulation of interferon-gamma production	21	2.71	6.90E-03
Regulation of interferon-gamma production	27	2.22	1.14E-02

### Immune cell differential methylation is strongly associated with differential gene expression

**Fig. 2:**
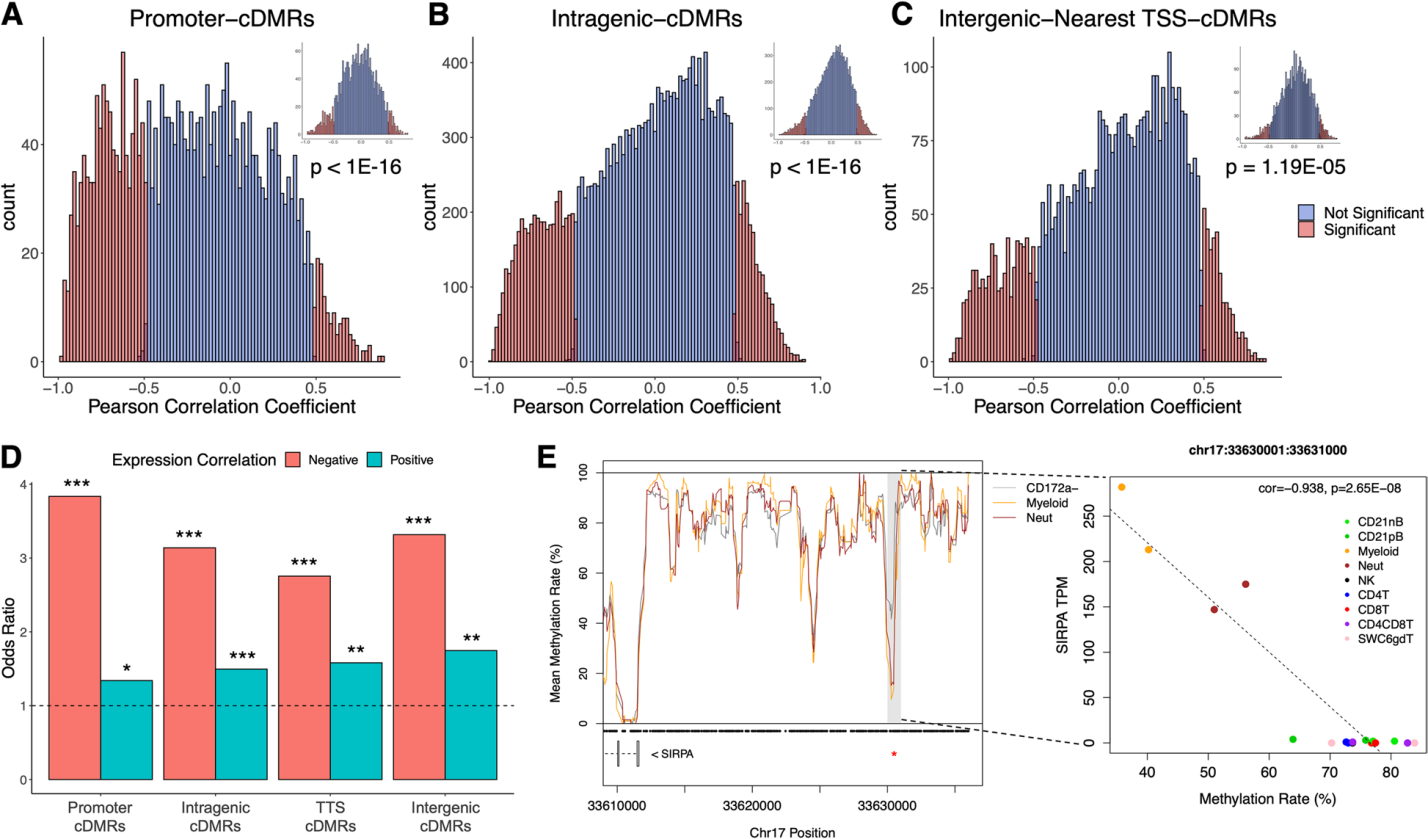
Porcine immune cell differential methylation is associated with differential gene expression. **A**-**C** Histograms of cDMR methylation-transcript abundance Pearson correlation coefficients across cell types in promoter, intragenic, and intergenic regions, inset by distributions at a random sampling of regions overlapping each feature. *p*-values indicate the significance in the shift to the left of the cDMR distribution relative to the random distribution as calculated by Wilcoxon rank sum tests. **D** Enrichment of cDMRs for regions positively and negatively correlated with gene expression, separated by gene feature. * = *p* < 0.05, ** = *p* < 1E-3, *** = *p* < 1E-10 from Fisher’s exact test. **E** A monocyte and neutrophil lowly methylated region located ~ 19 kb upstream of the *SIRPA* gene (red asterisk) is significantly negatively correlated with *SIRPA* abundance. Gray boxes indicate cDMRs, and black dots indicate CpG coordinates. cDMR = cell differentially methylated region

To determine if immune cell differential methylation was associated with local gene expression, we utilized bulk RNA-sequencing data generated from the same sorted porcine immune cell populations [18] and tested for significant correlations between cDMRs and transcript abundance of overlapping genes across all cell populations. Methylation rates of promoter and intragenic cDMRs were more likely to be significantly correlated with local transcript abundance compared to a random sampling of regions from each respective feature (Fig. 2A-B; Wilcoxon rank sum test *p* < 1E-16). For cDMRs in intergenic regions, we identified the gene with the most proximal TSS and saw a similar overrepresentation of significant correlations between intergenic methylation and gene expression as those for cDMRs and overlapping genes (Fig. 2C; Wilcoxon rank sum test *p* = 1.19E-05). cDMRs across gene features were more enriched for negative associations with transcript abundance, although a small but significant enrichment for positive associations was also evident (Fig. 2D). We observed that genes in which cDMR methylation was positively correlated with transcript abundance were more lowly expressed on average compared to genes with negative methylation-transcript abundance associations, and furthermore that these positively correlated cDMRs exhibited higher methylation rates on average (Figure S4).

Within immune cell marker genes, cDMRs exhibited methylation rates that were significantly negatively correlated with abundances of corresponding transcripts (Figure S5). Furthermore, by identifying the most proximal TSSs to intergenic cDMRs, we identified a region approximately 19 kb upstream of the *SIRPA* TSS that was lowly methylated in myeloid cell types (Fig. 2E). While there was no evidence of TSS-proximal differential methylation between *SIRPA*-expressing and non-expressing cell types, methylation at this upstream locus was significantly negatively correlated with *SIRPA* abundance (*r* = -0.94), suggestive of putative enhancer-like function that is unique to the myeloid cell lineage. Collectively, these results elucidate context-specific associations between DNA methylation and both proximal and distal gene expression, and highlight putative sites of transcriptional epigenetic gene regulation.

**Fig. 3:**
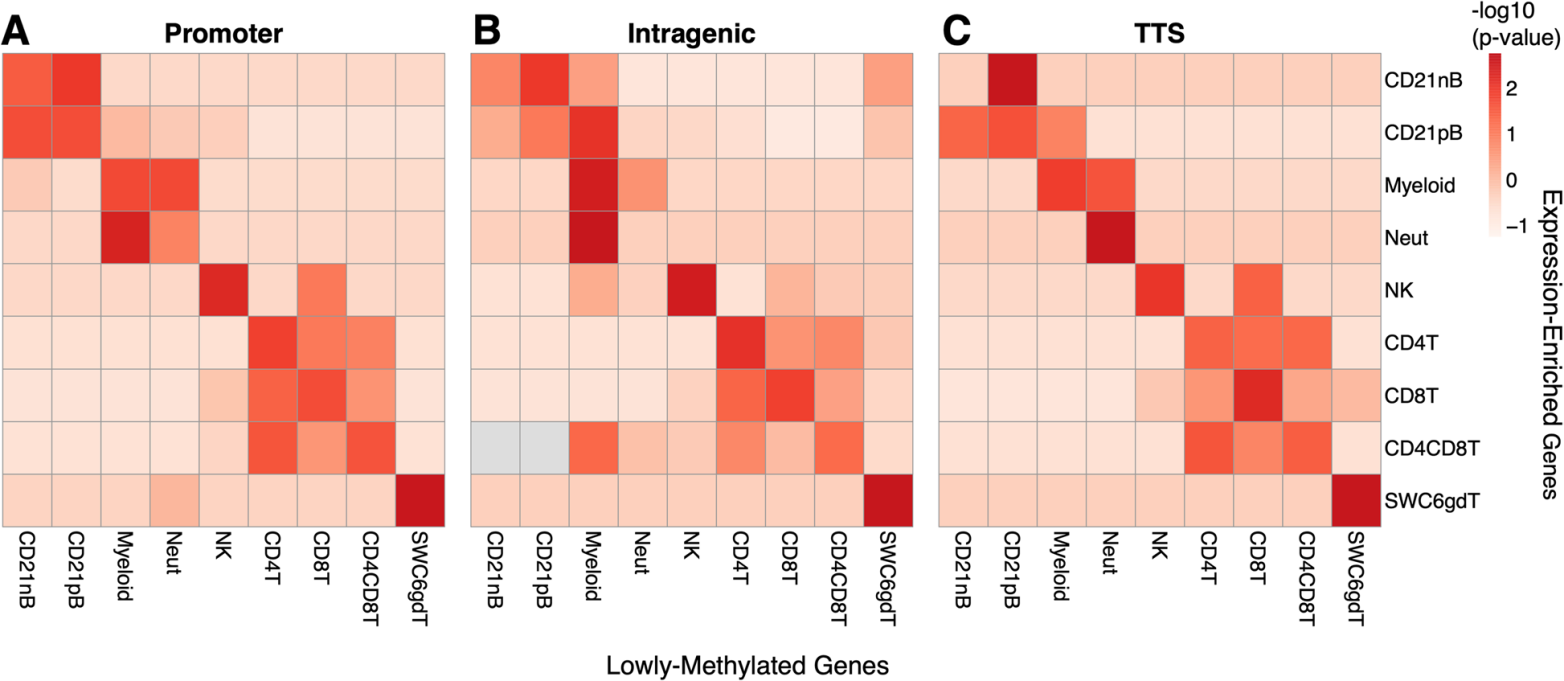
Cell lowly methylated regions (cLMRs) are enriched within expression-enriched genes. **A**-**C** Heatmap of normalized enrichment *p*-values between expression-enriched genes and genes overlapping (**A**) promoter, (**B**) intragenic, and (**C**) transcription termination site cLMRs for each cell type

We calculated the degree to which cLMRs associated with cell-enriched gene expression using the same RNA-seq data sets. We identified 2,895 genes exhibiting enriched gene expression in one or more cell types, ranging from 244 genes in CD4CD8T cells to 1,261 in myeloid cells (Table S4). Overall, cLMRs were highly overrepresented among expression-enriched genes of the same or related cell types (Fig. 3A-C). Promoter and TTS cLMRs tended to have stronger and exclusive associations with expression-enriched genes of the same cell type; however this pattern was also observed to a lesser extent among intragenic cLMRs, demonstrating that low methylation outside of promoter regions was associated with enriched gene expression. We provide here ample evidence that cell-specific differential methylation in porcine immune cells is highly correlated with transcript abundance and co-localizes with genes associated with immune cell state.

### Cell lowly methylated regions are enriched for cell-specific transcription factor binding motifs

**Fig. 4:**
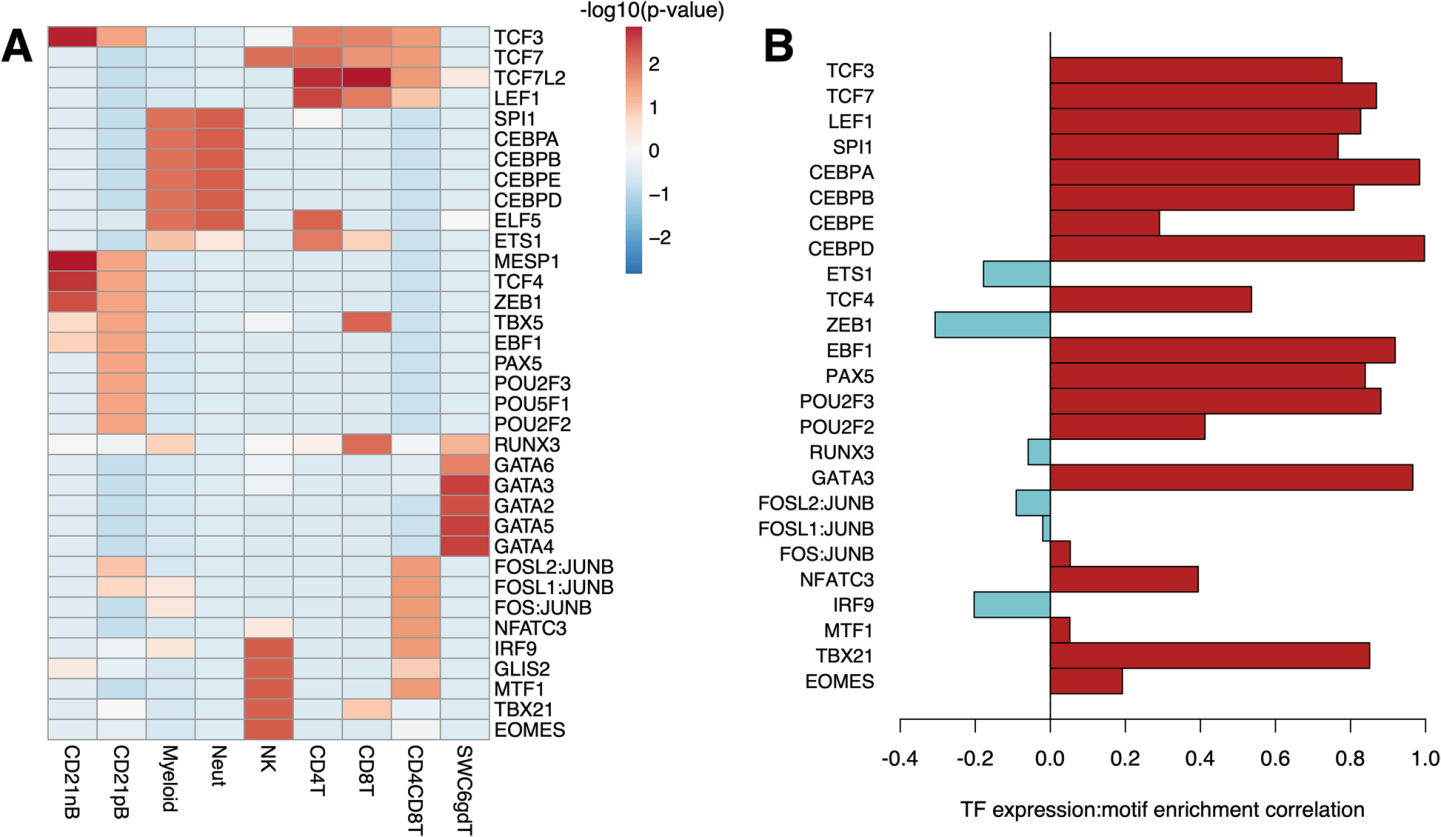
Porcine immune cell lowly methylated regions (cLMRs) co-localize with unique transcription factor (TF) binding motifs. **A** Normalized heatmap of cLMR enrichment scores for most significantly enriched TF binding motifs for each sorted immune cell population. **B** Pearson correlation coefficients between TF expression and –log10(*p*-value) of motif enrichment among cLMRs across cell types. Red and blue bars indicate positive and negative correlations, respectively

Because DNA methylation is known to inhibit TF binding [46], and lowly methylated regions are thus more likely to be permissive to TF binding, we submitted cLMR and random control sequences for analysis of motif enrichment to identify enriched TF binding motifs. Clustering of cells based on cLMR enrichment for 1,808 human motifs grouped cell types into B cells, myeloid cells, NK cells and CD4CD8T cells, and the remaining T cell subpopulations (Figure S6). The most enriched binding motifs among cLMRs were highly cell-type specific, and many play an important role in the respective cell type’s development, maturation, and function (Fig. 4A). These included motifs for transcription factor E2-alpha (TCF3) in lymphocytes and the CCAAT enhancer binding protein (CEBP) family in myeloid cells. Other binding motifs were enriched among lymphocyte subtypes: the early B cell factor 1 (EBF1) motif was enriched among B cell LMRs, while transcription factor 7 (TCF7) and lymphoid enhancer binding factor 1 (LEF1) motifs were enriched in αβ T cell (CD4T, CD8T, CD4CD8T) cLMRs. Several TFs possessed enriched motifs among cLMRs for a single cell type, such as T-bet (TBX21) and Eomesodermin (EOMES) among NK cLMRs, FOS:JUN heterodimers among CD4CD8T cLMRs, and GATA binding proteins (GATAs) among SWC6gdT cLMRs.

To determine if binding motif enrichment among cLMRs was associated with increased expression of the corresponding TFs, we assessed transcript abundances for all TFs in Fig. 4A. Of the 25 expressed TFs, 13 exhibited transcript abundances that were significantly positively correlated (*r* > 0.5) with cLMR enrichment for the corresponding binding motif, and all but six correlations were positive (Fig. 4B). Furthermore, many of the TFs exhibited significantly enriched expression in the same cell types for which their binding motifs were enriched among cLMRs: *EBF1*, *POU2F3*, *PAX5*, *TCF3*, and *TCF4* (B cells), *TCF7* and *LEF1* (T cells), *TBX21* (NK cells), *CEBPA/B/D* and *SPI1* (myeloid cells), and *GATA3* (SWC6gdT cells) (Figure S7). These data support the conclusion that regions of cell-specific low methylation are associated with regulatory potential by biologically-relevant trans-acting factors regulating immune cell development and function.

### Immune cell differential methylation co-localizes with candidate loci for immune capacity and disease traits

**Fig. 5:**
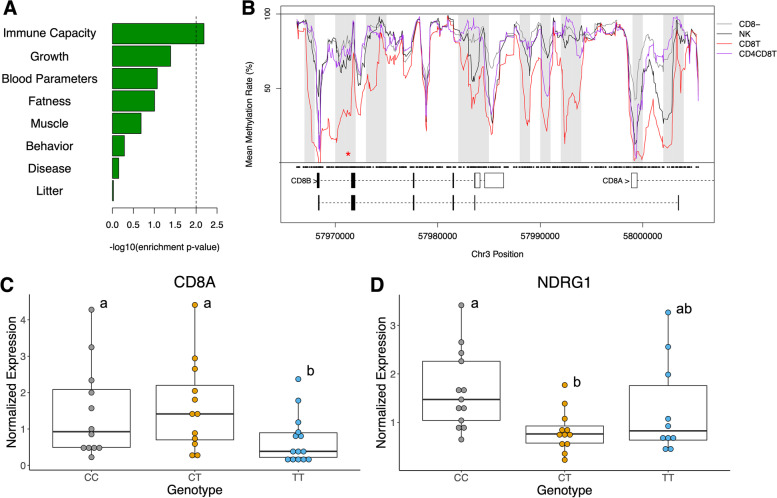
Immune cell differentially methylated region (cDMRs) harbor immune capacity GWAS SNPs. **A** Enrichment barplot of GWAS SNPs for various trait classes among cDMRs. Dashed line indicates threshold for significance. **B** *CD8B/CD8A* locus methylation plot including a CD8^+^ T cell LMR overlapping a CD8^+^ T cell %/CD8^−^ T cell % GWAS SNP (red asterisk). Gray boxes indicate cDMRs, and black dots indicate CpG coordinates. **C** Normalized *CD8A* abundance in porcine peripheral blood mononuclear cells by *CD8B* SNP genotype. CC: *N* = 12, CT: *N* = 12, TT: *N* = 14. **D** Normalized *NDRG1* abundance by *NDRG1* SNP genotype. CC: *N* = 13, CT: *N* = 12, TT: *N* = 10. Different letters indicate statistically significant differences (FDR < 0.05)

To determine if observed immune cell differential methylation was associated with genomic regions influencing economically important traits, we identified cDMRs harboring previously-reported GWAS SNPs in the Pig QTL Database. Among various trait classes, we identified cDMR enrichment exclusively for SNPs associated with immune capacity traits, and no significant enrichment for SNPs associated with growth, reproductive, or behavioral traits (Fig. 5A). Fifty-three immune capacity GWAS SNPs overlapped cDMRs, and several were within biologically-relevant genes exhibiting transcript abundance that was significantly correlated with regional methylation rate (Table 4). We identified a SNP associated with the blood cell type composition traits ‘CD8^+^ T cell percentage’ and ‘CD8^−^ T cell percentage’ that co-localized with a CD8T cLMR within *CD8B* (Fig. 5B) [47]. *CD8B* encodes the beta subunit of the CD8αβ T cell co-receptor and CD8T cells exhibited the highest *CD8B* transcript abundance (log2FoldChange = 6.32). The GWAS SNP (rs81371115, 4:g.57971247C > T) lies in an intronic CpG upstream of exon 2. To determine if this SNP was also associated with local gene expression, we quantified transcript abundances of *CD8B* and *CD8A* via RT-qPCR in leukocytes across rs81371115 genotypes in an unrelated pig resource population. While *CD8B* abundances were below the threshold of detection, *CD8A* abundance was significantly associated with SNP genotype (*p* = 0.027), with the TT genotype exhibiting significantly lower abundance than CC and CT genotypes (Fig. 5C). These results corroborate previous findings that rs81371115 genotype is associated with CD8T cell composition phenotypes, and demonstrate the regulatory potential of the LMR overlapping this SNP in influencing CD8 co-receptor expression.

**Table 4:** SNPs within cLMRs associated with transcript abundance

**SNP ID**	**SNP Pos**	**Trait**	**cLMR Cell**	**Gene**	**Meth-TPM cor**	**Meth-TPM** ***p*value**	**Expression Enrichment**
rs81368734 (T/C)	3:30,799,911	CD8 + T%, CD8-T%	CD4T, SWC6gdT	*SNX29*	0.673	0.003	CD21pB
rs81370925 (T/C)	3:56,530,168	CD8 + T%, CD8-T%, CD3-CD8-T%	CD21nB, SWC6gdT	*ZAP70*	-0.752	0.0005	CD21nB, CD8T, SWC6gdT, Neut
rs81371115 (C/T)	3:57,971,274		CD8T	*CD8B*	-0.839	2.56E-05	CD8T, NK
rs81371265 (G/A)	3:58,968,002		NK	*ATOH8*	-0.715	0.0012	CD8T, NK
rs81399335 (G/A)	8:30,368,512	Lymphocyte percentage, 20 d	CD21pB, Myeloid	*KLHL5*	-0.446	0.073	Myeloid
rs327164077 (T/C)	4:8,034,723	Number of mummified pigs	Myeloid, NK	*NDRG1*	-0.614	0.0087	Myeloid

Lastly, we also queried disease-associated GWAS SNPs for co-localization with cDMRs, and identified a myeloid and NK cell LMR in *N-Myc Downstream Regulated 1* (*NDRG1*) harboring a SNP associated with number of mummified piglets (rs327164077, 4:g.8034723 T > C). The phenotype of mummified piglets is often caused by porcine reproductive and respiratory syndrome virus (PRRSV) infection in the sow [48], and *NDRG1* has previously been implicated in PRRSV response [49]. In adult porcine leukocytes, we observed significantly lower *NDRG1* abundance in the CT genotype relative to CC (*p* = 0.001; Fig. 5D) while the TT genotype exhibited intermediate expression, providing evidence of a shared genetic association for reproductive performance and *NDRG1* expression. In summary, our results have enhanced functional annotation in genomic regions at which genetic variation is associated with immune traits, and have further linked such variation to local gene expression.

## Discussion

We report here the first genome-wide assessment of DNA methylation across sorted circulating porcine immune cell populations, and correlate methylation with gene expression of the same sorted cells. Using these data, we have identified thousands of genomic regions where methylation rate is associated with immune cell type, which suggests that these regions play important roles in cell-specific gene regulation. Previous ENCODE studies have surveyed DNA methylation in B cells as well as various T cell subpopulations from healthy individuals and leukemia patients, and have reported similar cell-specific DNA methylation patterns between cell types and states [20, 22]. We have surveyed a greater diversity of circulating immune cell populations in the pig derived from both myeloid and lymphoid lineages, including T cell populations that are uniquely overrepresented in the pig relative to human and mouse. Our DNA methylation analyses thus provide unique insights into epigenetic gene regulation in understudied immune cells.

We observed global DNA methylation rates between 80–84% that are comparable to those reported across mammalian species [50]. Among cell types, CD21pB, NK, and CD4CD8T cells possessed significantly lower methylation rates relative to other immune cells. Previous studies have shown that B and NK cell development and activation are associated with global DNA hypomethylation [51–54]. In addition, we identified significant correlates with global methylation rates, most notably abundance of DNA methyltransferase transcripts. While DNMT3A, DNMT1 and DNMT3B are all responsible for catalyzing cytosine methylation, transcript abundances of the latter two were negatively correlated with global CpG methylation rates in our study, such that their abundances subtracted from *DNMT3A* abundance explained the greatest proportion of variance in immune cell methylation rate. DNMT3A is responsible for de novo DNA methylation, thus higher expression would be expected to result in increased methylation rates genome-wide [55]. The negative correlations between global methylation and *DNMT1* and *DNMT3B* abundance may be due to associations with replicating DNA and dividing cells. Newly synthesized DNA is inherently hemimethylated following replication, and requires the activity of maintenance methyltransferase *DNMT1* to conserve CpG methylation [56]. Furthermore *DNMT3B*, a de novo methyltransferase, can promote mitotic division by methylating centromeric regions and establishing chromosome stability [57]. We provide evidence that global hypomethylation of cell types may be associated with increased rates of cell division due to their higher rates of relative centromeric methylation as well as increased expression of genes involved in DNA replication and cell division. Overall, these data suggest that variation in genome-wide DNA methylation levels can be explained in part by differences in *DNMT* expression and, potentially more directly, mitotic capacity of cell types.

We identified tens of thousands of genomic loci exhibiting differential methylation associated with porcine immune cell type. As regions of epigenomic variation, cDMRs represent likely regions of gene regulation that are associated with cell identity and function. In support of this claim, clustering by cDMR methylation rate broadly grouped samples by cell lineage, highlighting the extent of methylation conservation between related cell types. Numerous cDMRs were present within immune cell-specific co-receptors. The majority of cDMRs were further classified as cLMRs for the cell types known to express the encoded marker, suggesting that these loci may promote expression of the resulting transcript. While cLMRs of some marker genes were limited to promoter regions (e.g. *CD4*, *CD19*), others exhibited cell-specific low methylation in intragenic regions (e.g. *CD8A*, *SIRPA*) which is suggestive of intronic enhancer elements that have previously been shown to be widespread in human tissues and cell lines [20]. We also observed instances of discordance between marker gene methylation and expression, namely at the *CD8A* locus where methylation in CD4T cells was similar to that of CD4CD8T and other CD8α^+^ cell types despite absence of *CD8A* expression. These findings suggest that CD4T cells may be ‘primed’ for *CD8A* expression via hypomethylation, and support the hypothesis that peripheral porcine CD4T cells readily gain *CD8A* expression to become CD8CD4 double-positive T cells [45]. Among cell lowly methylated genes, we identified enrichment for cell-specific processes for the majority of cell types, demonstrating that low methylation genome-wide occurs disproportionately in functionally-relevant loci.

By integrating methylation data with transcript abundance data from the same porcine immune cell samples, we identified an overrepresentation of regions at which methylation rate was significantly correlated with expression of overlapping genes. Furthermore, intergenic cDMRs were disproportionately correlated with abundance of the most proximal genes transcript, signifying putative roles for these regions as distal regulators of gene expression. The majority of significant cDMR-transcript abundance correlations—particularly in promoter regions—were negative, which is in agreement with the predominantly repressive role of DNA methylation at promoter and enhancer regions previously reported in other mammals [20, 25]. However, positive correlations between cDMR methylation and gene expression were also observed, and were more common in genes exhibiting lower expression and higher methylation. This finding is in agreement with previous work reporting expression-dependent relationships between differential gene methylation and transcript abundance: for genes of lower basal expression levels, methylation increases with increasing transcription rates due to increased chromatin opening by RNA Polymerase II, but at a certain expression threshold methylation disrupts RNA Pol II and vice versa [58]. We demonstrate that integrating immune cell methylation data with gene expression data can identify putative distal-acting regulatory elements, exemplified by a putative myeloid enhancer upstream of *SIRPA* at which methylation was negatively correlated with *SIRPA* transcript abundance. Recent ATAC-seq data from the same myeloid cell populations has identified a region of open chromatin in the same 1 kb interval (JHU, RJC and CKT, unpublished results), providing additional evidence for a myeloid-specific enhancer. Cell-enriched gene expression was most strongly correlated with lowly methylated regions, suggesting that any active role DNA methylation plays in regulating porcine immune gene expression is largely suppressive. The colocalization of enriched genes with cLMRs across all gene features signifies the importance of intragenic epigenetic gene regulation in maintaining cell-type specific gene expression.

We have shown that immune cell differential methylation is associated with regions harboring TF binding motifs, many of which govern cell-specific functions. It has long been understood that differential methylation functions in coordination with changes in trans-acting factor binding to regulate gene expression, although the exact mechanisms governing this relationship remain debated and are likely context-specific [46]. In general, DNA hypomethylation in promoters and enhancers is associated with increased binding of activating TFs, while hypermethylation is associated with exclusion of activating TFs and recruitment of transcriptional repressors [19, 21]. Human and mouse DNA methylation studies report enrichment for binding sites of functionally-relevant TFs within LMRs unique to tissues and cell types [22, 25, 26]. Similarly, we identified enriched TF motifs among cLMRs that play known roles in regulating development and function, including: CEBP family of TFs in myeloid cells [59–61]; EBF1 in B cells [62, 63], TCF7 in T cells [64], and EOMES and T-bet/TBX21 in NK cells [65]. Furthermore, we identified overrepresented motifs within porcine-enriched T cell LMRs that provide unique insight into gene regulation in these subpopulations. Multiple GATA TF motifs were enriched solely among SWC6gdT cLMRs in our analysis, and, among these, *GATA3* exhibited enriched expression relative to other cell types in our study (log2FoldChange = 4.89). It is thus likely that GATA3 plays an important role in mature γδ T cells that is unique from its role in other T cell subtypes. A recent single-cell RNA-sequencing analysis in porcine PBMCs reported greater *GATA3* expression in CD2^−^ γδ T cell clusters relative to CD2^+^ γδ T cell clusters, suggesting even further restriction of GATA3 activity to certain γδ T cell subpopulations that is also supported by protein data [66]. In CD4CD8T cLMRs, we identified enrichment of multiple AP-1 TF complex motifs, with c-Fos:JunB heterodimers being the most enriched. *c-fos* deficiency in mice has previously been linked to decreased AB T cell production in cultured thymocytes [67]. However, the presence of lowly methylated AP-1 binding sites in mature DP T cells indicates a potential outsized role for these TFs beyond thymic T cell development. AP-1 TF binding has been shown to activate IFNγ, which CD4CD8T cells are known to express at high levels [68]. Future studies should seek to assess the genome-wide consequences of AP-1 motif hypomethylation and its association with AP-1 activity in porcine DP T cells.

Lastly, we provide evidence that differential methylation is strongly associated with reported QTL for pig immune traits. Previous studies have identified tissue- and cell-specific enrichment of LMRs for human and livestock GWAS SNPs of relevant traits [22–24]. We similarly identified immune cDMR enrichment exclusively for pig immune capacity traits, and not for trait classes that are less likely to be directly impacted by immune system gene regulation. In addition to observed overall enrichment, cDMRs overlapped GWAS SNPs within strong candidate genes for associated traits. These included a SNP associated with various T cell subpopulation percentages in a CD8T cLMR overlapping the *CD8B/CD8A* locus. We show here that the same SNP was associated with leukocyte *CD8A* expression in a separate pig population; however due to the undetermined composition of assayed leukocyte samples we cannot directly link this SNP to variation in *CD8A* transcription as opposed to altered abundances of each cell type in the sample. We also show that a SNP within a myeloid and NK cell LMR overlapping *NDRG1* is significantly associated with *NDRG1* expression, while also having been previously associated with number of mummified piglets [69]. Both reproductive performance and *NDRG1* expression are negatively impacted by PRRSV infection, with downregulation of *NDRG1* correlated to increased PRRSV replication rate [48, 49]. It is thus intriguing to hypothesize that *NDRG1* expression may act as an intermediate molecular phenotype linking *NDRG1* genotype with observed reproductive variation, and that this relationship may be further affected by viral infection.

In summary, we have vastly improved the understanding of epigenetic gene regulation in the porcine immune system, and have contributed to current pig FAANG efforts through the generation of DNA methylation atlases in diverse immune cell types. We believe these data will prove valuable in further understanding how cell-specific epigenetic modifications contribute to pig immune and disease phenotypes, as well as inspire future studies seeking to integrate functional annotation data to enhance the search for causative variants for complex traits.

## Supplementary Information

**Additional file 1: Table S1.** Summary of cell differentially methylated regions (cDMRs).

**Additional file 2: Table S2.** Genes exhibiting low methylation in a single cell type.

**Additional file 3: Table S3I.** Enriched GO terms for cLMR genes.

**Additional file 4: Table S4.** Summary of gene expression enrichment in sorted porcine immune cells.

**Additional file 5: Figure S1.** Associations between immune cell global methylation and DNA methyltransferase (DNMT) expression. Moderate negative and positive correlations were observed between global methylation and normalized transcript abundance of DNMT1 (**A**) and DNMT3A (**B**), respectively. Normalized DNMT3B abundance was significantly negatively correlated with global DNA methylation (**C**). Considering abundance of all DNMTs, the normalized abundances of DNMT1 and DNMT3B subtracted from DNMT3A abundance were significantly positively correlated with global methylation (**D**). **Figure S2.** Methylation rates across immune cell marker genes *CD19* (**A**), *SIRPA* (**B**), and *TBX21* (**C**), and *CD4* (**D**) for expressing and non-expressing cell types. Gray boxes indicate cell differentially methylated regions (cDMRs) within each gene locus, and black dots indicate CpG coordinates. **Figure S3.** (**A**) Dot plot of average methylation at CD8A TSS-proximal cell differentially methylated regions (cDMRs). (**B**) Dot plot of CD8A transcript abundance as percentage of maximum transcripts per kilobase million (TPM) across samples. Letters indicate statistically significant differences between means (*p* < 0.05). **Figure S4.** Positive methylation:expression correlations are associated with more lowly expressed genes and highly methylated regions. (**A**) Transcripts per kilobase million (TPM) of genes overlapping cDMRs negatively and positively correlated with gene expression, separated by gene feature. (**B**) Methylation levels of cDMRs negatively and positively correlated with gene expression, separated by gene feature. **=*p*<0.05, ***=*p*<1E-05 from Wilcoxon rank sum test for significant shift in distribution of TPM/methylation between cDMRs negatively and positively correlated with gene expression. NS=not significant. **Figure S5.** Immune cell co-receptor gene cDMRs are significantly negatively correlated with transcript abundance. Scatter plots of methylation rates at (**A**) *CD4* promoter, (**B**) *CD8A* TTS, and (**C**) *CD19* promoter and corresponding transcript abundance across porcine immune cells. **Figure S6.** Clustering of cell types based on cell lowly methylated region (cLMR) normalized enrichment for 1,808 motifs in the vertebrates (in vivo and in silico) database. **Figure S7.** Transcript abundance of five transcription factors with cLMR-enriched binding motifs: (**A**) EBF1, (**B**) CEBPA, (**C**) TBX21, (**D**) TCF7, and (**E**) GATA3. *** = Significantly enriched gene expression.

## Data Availability

The datasets generated and analyzed during the current study are available in the European Nucleotide Archive (ENA) repository (https://www.ebi.ac.uk/ena/browser/view/PRJEB47517?show=reads and study accession number PRJEB47517). Myeloid ATAC-seq data referenced in the discussion is available in the ENA repository (https://www.ebi.ac.uk/ena/browser/view/PRJEB51699?show=reads and study accession number PRJEB51699). Code used to analyze WGBS data can be found at https://github.com/rjcorb.
