# Supplementary material for: Assessment of DNA methylation in porcine immune cells reveals novel regulatory elements associated with cell-specific gene expression and immune capacity traits
Source: BMC Genomics. 2022 Aug 11;23:575. doi: 10.1186/s12864-022-08773-5 (PMC9367135; doi:10.1186/s12864-022-08773-5)
Supplement: Supplementary file 5 — Additional file 5: Figure S1. Associations between immune cell global methylation and DNA methyltransferase (DNMT) expression. Moderate negative and positive correlations were observed between global methylation and normalized transcript abundance of DNMT1 (A) and DNMT3A (B), respectively. Normalized DNMT3B abundance was significantly negatively correlated with global DNA methylation (C). Considering abundance of all DNMTs, the normalized abundances of DNMT1 and DNMT3B subtracted from DNMT3A abundance were significantly positively correlated with global methylation (D). Figure S2. Methylation rates across immune cell marker genes CD19 (A), SIRPA (B), and TBX21 (C), and CD4 (D) for expressing and non-expressing cell types. Gray boxes indicate cell differentially methylated regions (cDMRs) within each gene locus, and black dots indicate CpG coordinates. Figure S3. (A) Dot plot of average methylation at CD8A TSS-proximal cell differentially methylated regions (cDMRs). (B) Dot plot of CD8A transcript abundance as percentage of maximum transcripts per kilobase million (TPM) across samples. Letters indicate statistically significant differences between means (p < 0.05). Figure S4. Positive methylation:expression correlations are associated with more lowly expressed genes and highly methylated regions. (A) Transcripts per kilobase million (TPM) of genes overlapping cDMRs negatively and positively correlated with gene expression, separated by gene feature. (B) Methylation levels of cDMRs negatively and positively correlated with gene expression, separated by gene feature. **=p<0.05, ***=p<1E-05 from Wilcoxon rank sum test for significant shift in distribution of TPM/methylation between cDMRs negatively and positively correlated with gene expression. NS=not significant. Figure S5. Immune cell co-receptor gene cDMRs are significantly negatively correlated with transcript abundance. Scatter plots of methylation rates at (A) CD4 promoter, (B) CD8A TTS, and (C) CD19 promoter and [file 12864_2022_8773_MOESM5_ESM.pdf]

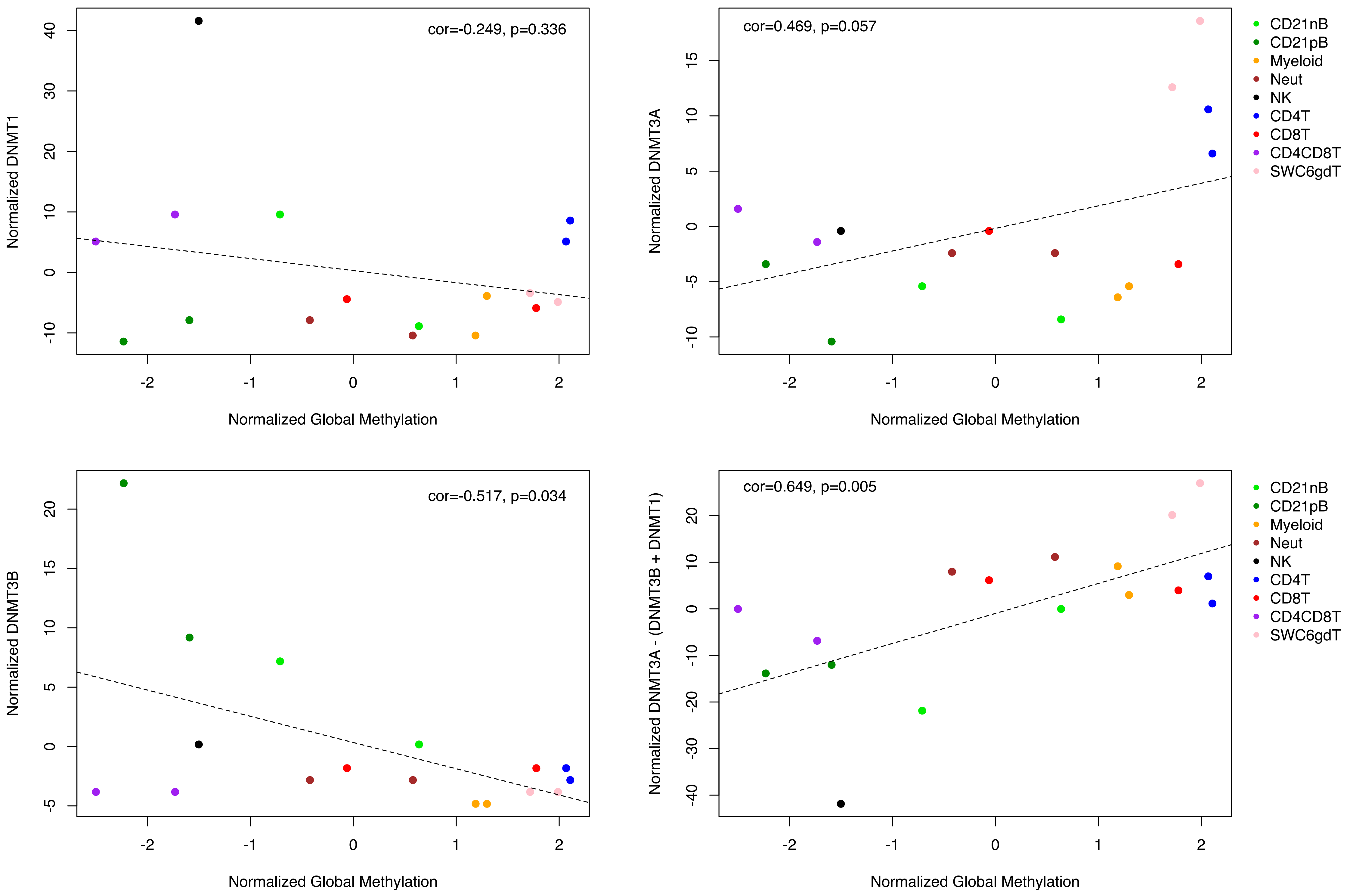

Figure S1. Associations between immune cell global methylation and DNA methyltransferase (*DNMT*) expression. Moderate negative and positive correlations were observed between global methylation and normalized transcript abundance of *DNMT1* **(A)** and *DNMT3A* **(B)**, respectively. Normalized *DNMT3B* abundance was significantly negatively correlated with global DNA methylation **(C)**. Considering abundance of all DNMTs, the normalized abundances of *DNMT1* and *DNMT3B* subtracted from *DNMT3A* abundance were significantly positively correlated with global methylation **(D)**.

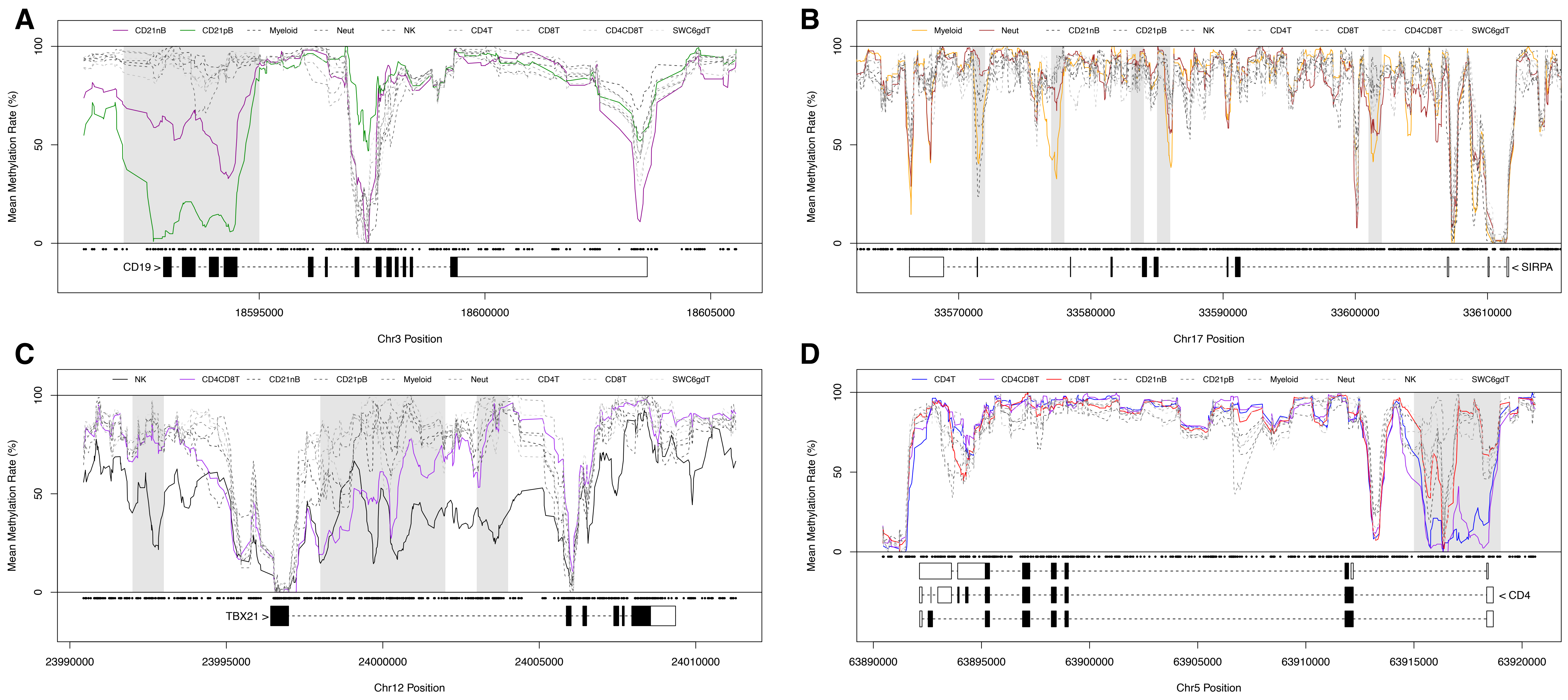

Figure S2. Methylation rates across immune cell marker genes *CD19* **(A)**, *SIRPA* **(B)**, and *TBX21* **(C)**, and *CD4* **(D)** for expressing and non-expressing cell types. Gray boxes indicate cell differentially methylated regions (cDMRs) within each gene locus, and black dots indicate CpG coordinates.

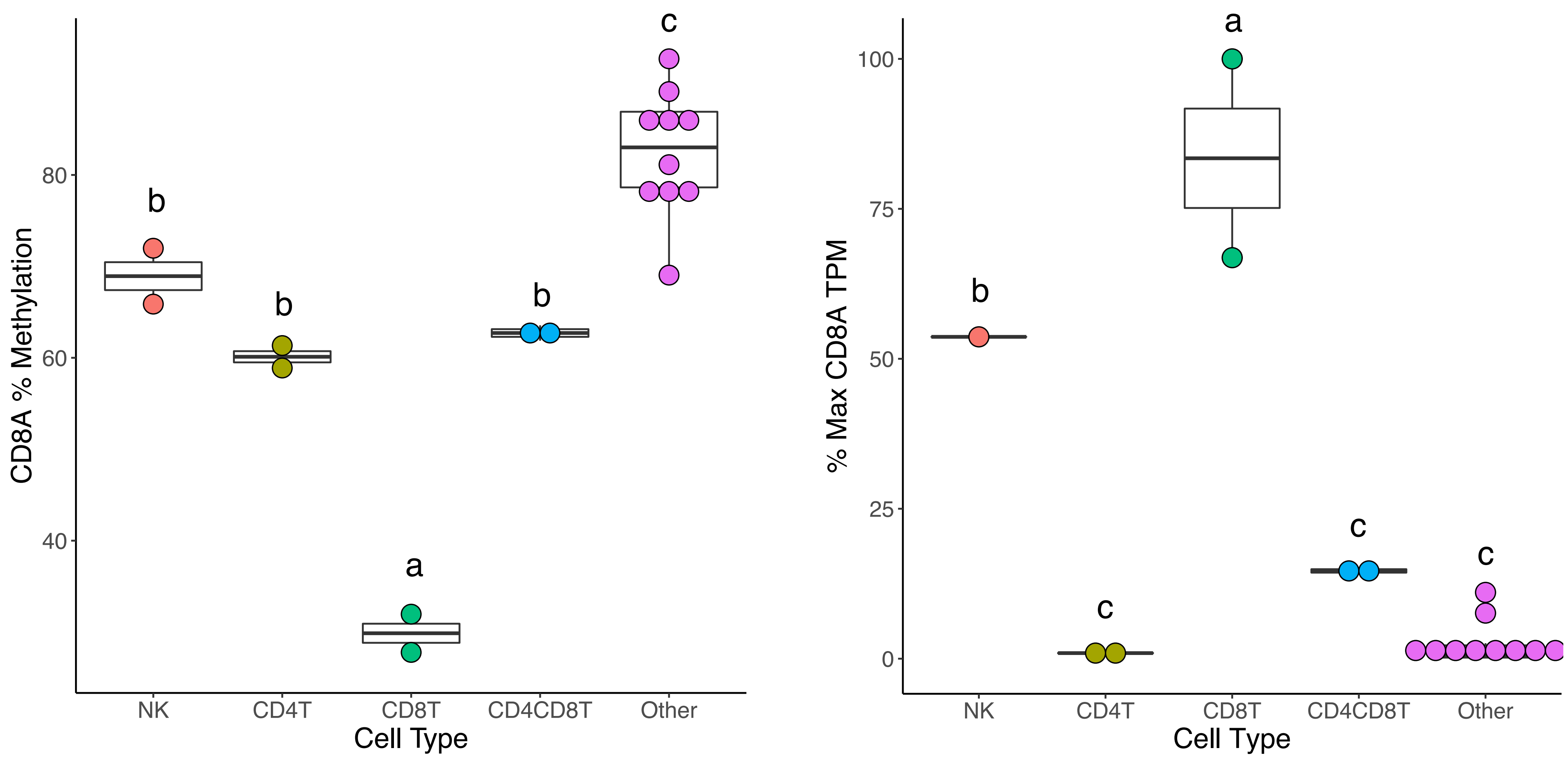

Figure S3. (A) Dot plot of average methylation at *CD8A* TSS-proximal cell differentially methylated regions (cDMRs). **(B)** Dot plot of *CD8A* transcript abundance as percentage of maximum transcripts per kilobase million (TPM) across samples. Letters indicate statistically significant differences between means ($p < 0.05$).

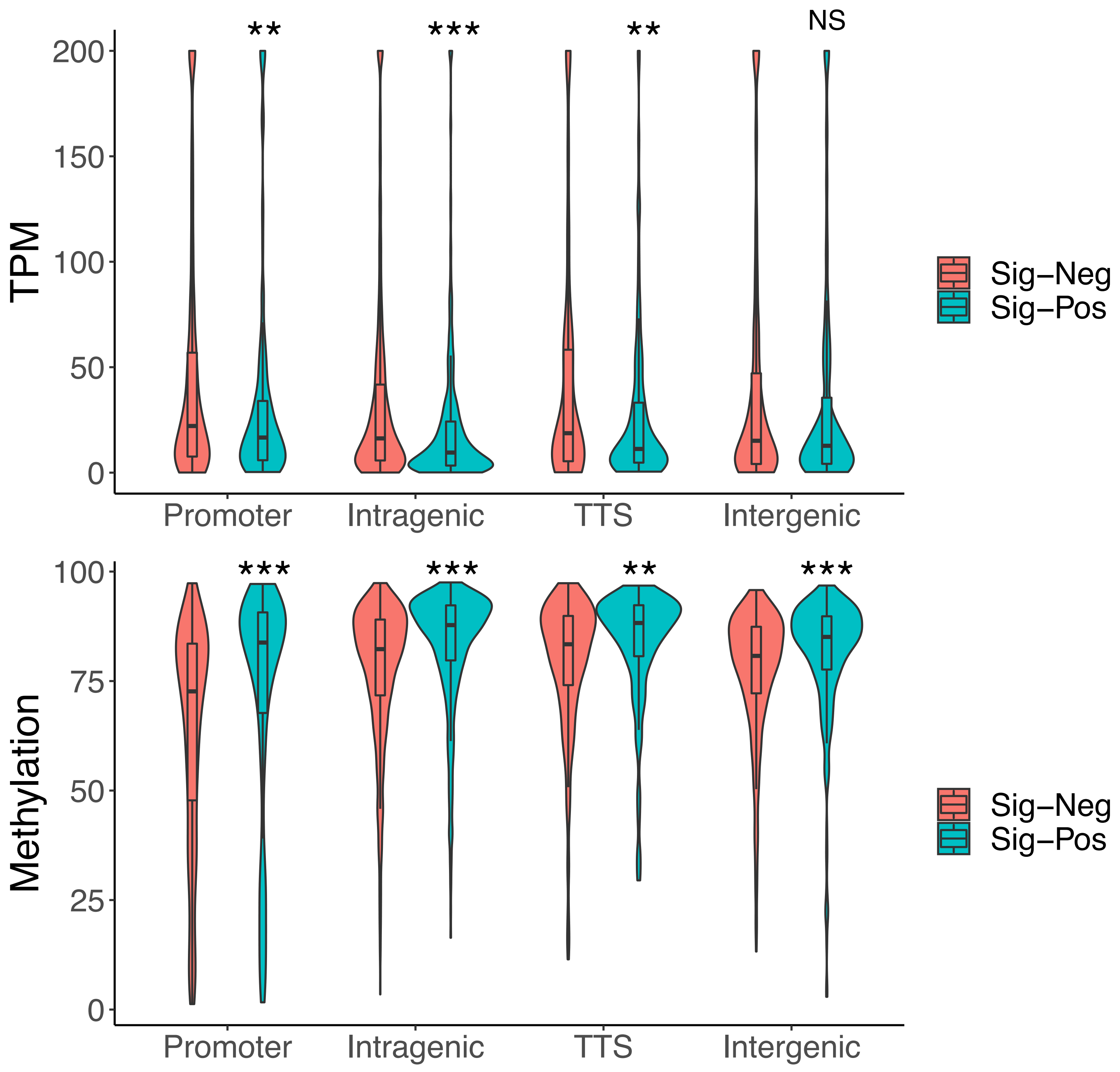

Figure S4. Positive methylation:expression correlations are associated with more lowly expressed genes and highly methylated regions. (A) Transcripts per kilobase million (TPM) of genes overlapping cDMRs negatively and positively correlated with gene expression, separated by gene feature. **(B)** Methylation levels of cDMRs negatively and positively correlated with gene expression, separated by gene feature. **=p<0.05, ***=p<1E-05 from Wilcoxon rank sum test for significant shift in distribution of TPM/methylation between cDMRs negatively and positively correlated with gene expression. NS=not significant.

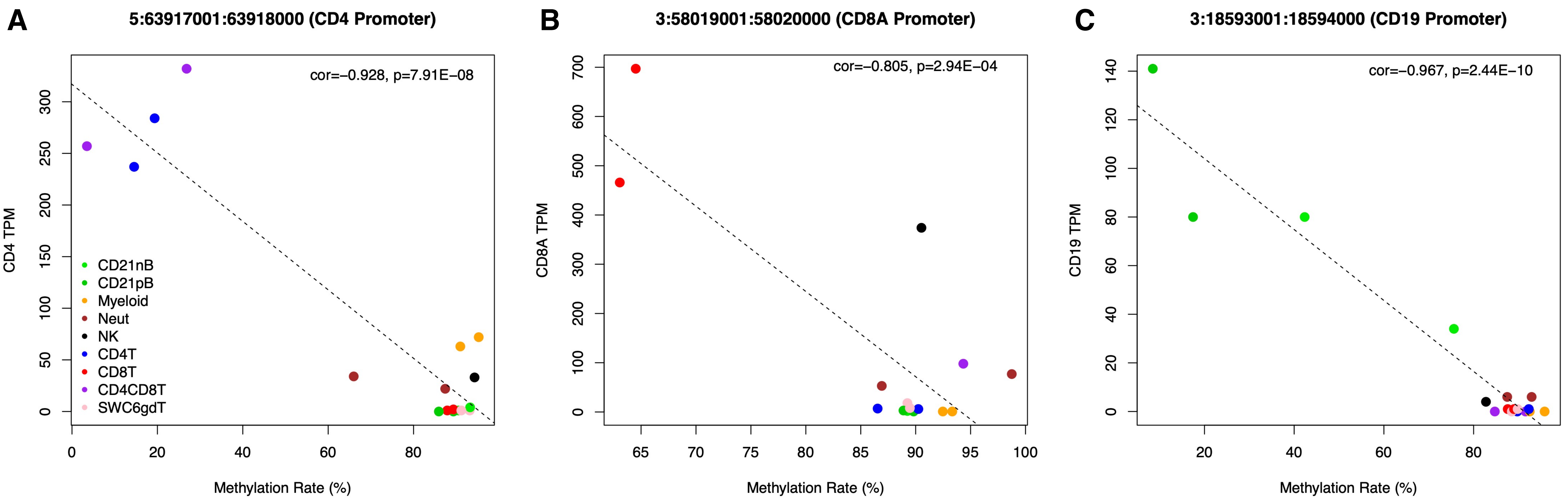

Figure S5. Immune cell co-receptor gene cDMRs are significantly negatively correlated with transcript abundance. Scatter plots of methylation rates at **(A)** *CD4* promoter, **(B)** *CD8A* TTS, and **(C)** *CD19* promoter and corresponding transcript abundance across porcine immune cells.

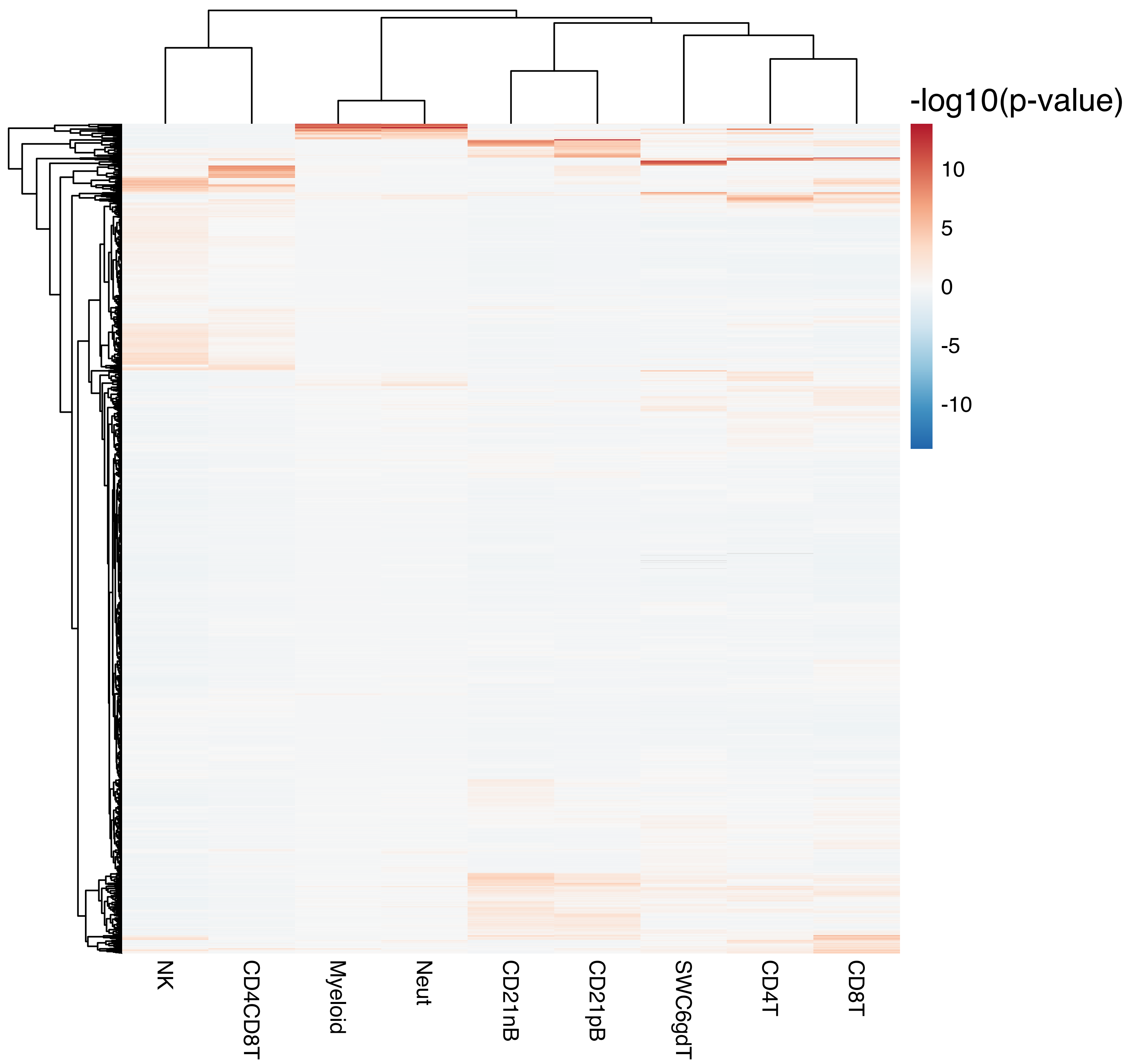

Figure S6. Clustering of cell types based on cell lowly methylated region (cLMR) normalized enrichment for 1,808 motifs in the vertebrates *(in vivo and in silico)* database.

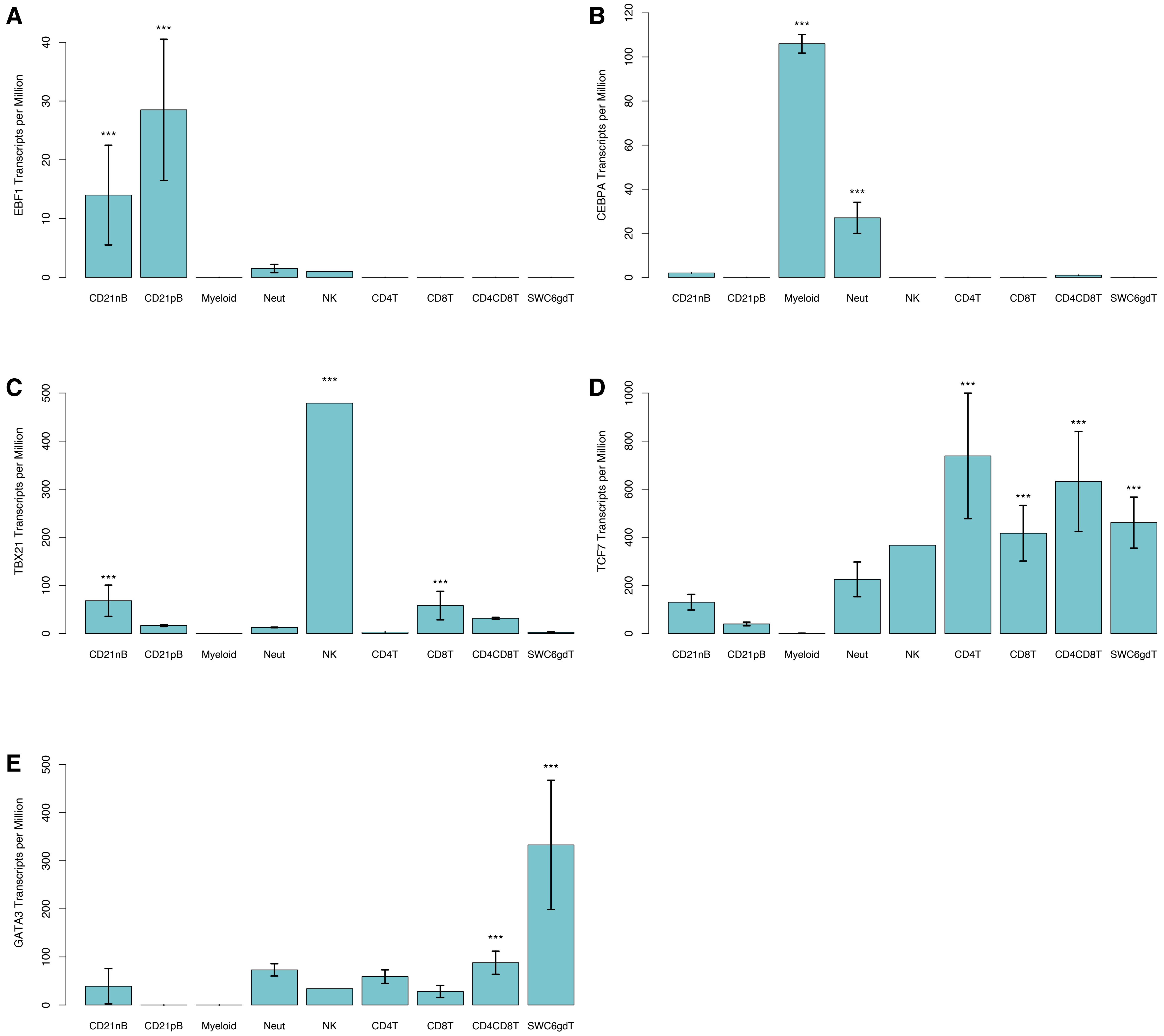

Figure S7. Transcript abundance of five transcription factors with cLMR-enriched binding motifs: **(A)** *EBF1*, **(B)** *CEBPA*, **(C)** *TBX21*, **(D)** *TCF7*, and **(E)** *GATA3*. *** = Significantly enriched gene expression.
